# Expressions of Cushing’s syndrome in multiple endocrine neoplasia type 1

**DOI:** 10.3389/fendo.2023.1183297

**Published:** 2023-06-20

**Authors:** William F. Simonds

**Affiliations:** Metabolic Diseases Branch, National Institute of Diabetes and Digestive and Kidney Diseases, National Institutes of Health, Bethesda, MD, United States

**Keywords:** familial neoplasia syndrome, ACTH - independent CS, Cushing's adenoma, pituitary tumor, MEN1 = multiple endocrine neoplasia Type 1, corticotropinoma

## Abstract

Cushing’s syndrome (CS) resulting from endogenous hypercortisolism can be sporadic or can occur in the context of familial disease because of pituitary or extra-pituitary neuroendocrine tumors. Multiple endocrine neoplasia type 1 (MEN1) is unique among familial endocrine tumor syndromes because hypercortisolism in this context can result from pituitary, adrenal, or thymic neuroendocrine tumors and can therefore reflect either ACTH-dependent or ACTH-independent pathophysiologies. The prominent expressions of MEN1 include primary hyperparathyroidism, tumors of the anterior pituitary, gastroenteropancreatic neuroendocrine tumors, and bronchial carcinoid tumors along with several common non-endocrine manifestations such as cutaneous angiofibromas and leiomyomas. Pituitary tumors are present in about 40% of MEN1 patients, and up to 10% of such tumors secrete ACTH that can result in Cushing’s disease. Adrenocortical neoplasms occur frequently in MEN1. Although such adrenal tumors are mostly clinically silent, this category can include benign or malignant tumors causing hypercortisolism and CS. Ectopic tumoral ACTH secretion has also been observed in MEN1, almost exclusively originating from thymic neuroendocrine tumors. The range of clinical presentations, etiologies, and diagnostic challenges of CS in MEN1 are reviewed herein with an emphasis on the medical literature since 1997, when the MEN1 gene was identified.

## Introduction

**Figure 1:**
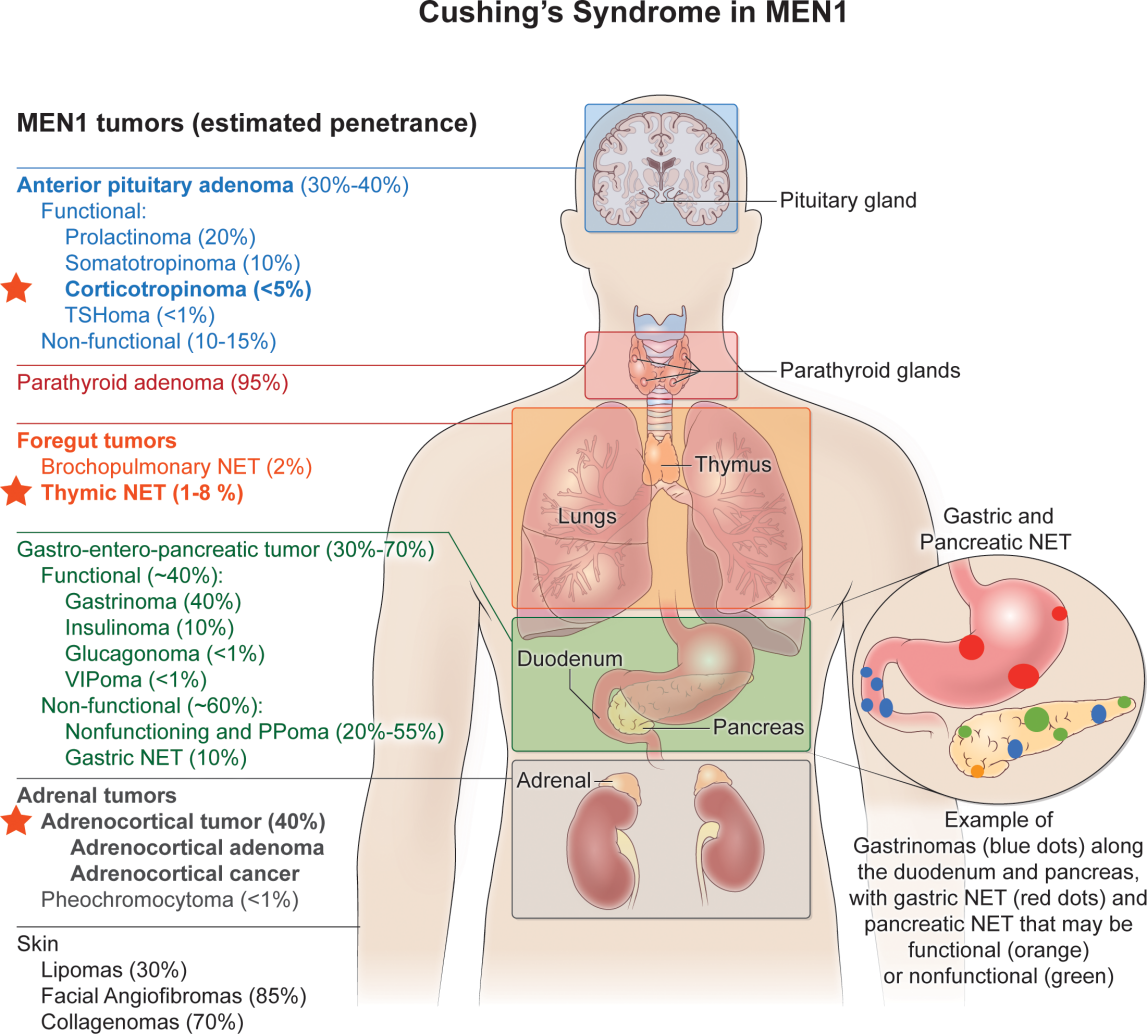
Cushing’s syndrome in MEN1. Multiple endocrine neoplasia type 1 (MEN1) is distinctive among familial endocrine tumor syndromes since Cushing’s syndrome in MEN1 can result from both ACTH-dependent and ACTH-independent causes. The former category includes Cushing’s disease (CD) and ectopic tumoral ACTH secretion, and the latter includes adrenocortical adenomas or carcinomas that secrete cortisol. The red stars indicate the MEN1-associated endocrine tumors that have been associated with either ACTH-dependent (pituitary corticotropinoma; thymic NETs) or ACTH-independent (adrenocortical adenoma or cancer) Cushing’s syndrome. See text for further details. Endocrine and non-endocrine tumors typical of MEN1 are listed on the left, with their estimated penetrance given in the parentheses. MEN1-associated endocrine tumors that can cause Cushing’s syndrome are in bold font. TSHoma, thyrotropinoma; NET, neuroendocrine tumor; PPoma, endocrine tumor that secretes pancreatic polypeptide.

Endogenous Cushing’s syndrome (CS) can occur either sporadically or else within families as a result of pituitary or nonpituitary neuroendocrine tumors (1). Among familial endocrine tumor syndromes, multiple endocrine neoplasia type 1 (MEN1) is distinctive because CS in MEN1 can result from both ACTH-dependent and ACTH-independent causes (**Figure 1**). The former category includes Cushing’s disease (CD) and ectopic tumoral ACTH secretion.

MEN1 is an autosomal dominant familial cancer predisposition syndrome whose most prominent expressions include multiglandular primary hyperparathyroidism, anterior pituitary tumors, foregut carcinoid tumors, and tumors of gastroenteropancreatic neuroendocrine cells (2). Anterior pituitary tumors occur in about half of MEN1 patients; and a small fraction of these secrete ACTH (3). Both non-functional and functional adrenocortical tumors occur in MEN1, and among these are included benign and malignant functional tumors causing CS (4). Ectopic CS from ACTH-secreting thymic carcinoids has also been reported in MEN1 (5–8). The MEN1 syndrome also includes non-endocrine tumors in several tissues (9).

This monograph provides a review and update on the range of clinical presentations, etiologies, and diagnostic challenges of CS in MEN1 patients, emphasizing the medical literature over the last two and a half decades since the cloning of the MEN1 gene (10).

## Cushing’s disease in MEN1

Tumors of the anterior pituitary tumor occur in about 30 to 40% of MEN1 patients. Approximately 4-10% of these pituitary tumors secrete ACTH (3, 11–13). Such tumors can secrete only ACTH or be pluri-hormonal. At the time of presentation, the pituitary tumors in MEN1 patients with CD may be either macro- or microadenomas. Which presentation may depend on how early in the disease course patients develop symptoms and seek medical attention. Another factor determining the size at initial detection of CD-associated pituitary adenomas is the extent and frequency of tumor surveillance in patients known to have, or to be at risk for, MEN1. Only a minority of MEN1 patients with CD develop visual symptoms or cavernous sinus invasion (12).

Although rare in sporadic CD, the presence of multiple synchronous or metachronous pituitary tumors may be discovered during the course of evaluation and/or surgery for CD in patients with MEN1. In a retrospective review of a large series of MEN1 patients that included 19 patients with CS, three of 11 MEN1 patients with CD (27%) had additional non-ACTH secreting pituitary microadenomas identified at surgery (14). This represented an incidence of multiple pituitary adenomas significantly higher than both that reported previously in a series of unselected sporadic pituitary adenomas (3 out of 116; 2.6%) (15) and that found in a large series of mainly sporadic CD cases (11 out of 658 total, excluding two MEN1 patients; approx. 2%) (16). Others have reported the presence of multiple synchronous or metachronous pituitary tumors in MEN1 patients both with and without CD (17–19).

Cushing’s disease can present in childhood or adolescence as the initial manifestation of MEN1 (**Table 1**) (20–22). The presenting signs and symptoms frequently include growth delay or retardation, weight gain and truncal obesity, headaches, and plethora. In females, hirsutism or menstrual irregularities may also be present at the time of diagnosis. Biochemical manifestations of hypercortisolism and pituitary imaging and localization studies resemble those of adults with CD (20–22).

**Table 1:** Cushing’s disease as the presenting manifestation of MEN1.

Patient	Age	Gender	Clinical presentation	MEN1 mutation	Year	Reference
1	13	M	Growth retardation, truncal obesity, muscle weakness	Y351H	2004	Rix et al. (20)
2	11	F	Weight gain, decreased growth rate, dyspnea on exertion	Y351H	2004	
3	12	F	Progressive obesity, growth retardation, short stature	L444P	2004	
4	11	M	NA	R460X	2004	Matsuzaki et al (21)
5	11	M	Weight gain, height arrest, irritability	Deletion of exons 1-2	2018	Makri et al. (22)
6	15	M	Weight gain, headaches, bone aches, stretch marks	R415X	2018	
7	9	M	Weight gain, height arrest, headaches, facial plethora	Q398X	2018	
8	17	F	Weight gain, facial plethora, facial hirsutism, menstrual irregularities	V19L	2018	
9	11	F	Weight gain, headaches	c.307delC frameshift	2018	
10	16	F	Weight gain, height arrest, headaches, muscle weakness, hirsutism	c.251-252delCT, S84Y frameshift	2018	

M, male; F, female; NA, not available

NA, not available.

In a large study of pediatric and adolescent patients with pituitary adenomas screened for predisposing germline mutations, two adolescents with recurrent or difficult-to-treat CD were found to harbor germline MEN1 mutations among 74 subjects with CD (23). In a large clinical and molecular analysis of 235 younger patients with apparently sporadic pituitary adenomas, two patients who presented with CD in their 20s were identified with the same germline variant of unknown significance in the MEN1 gene (24). It is controversial whether this particular variant, that encodes a R171Q change in the menin protein coding sequence, should be considered a polymorphism or mutation (25). A retrospective study of 80 children and adolescents with MEN1 included 18 patients with pituitary adenomas but none was found to have CD (26).

Somatic mutation of the MEN1 gene in sporadic pituitary tumors appears to be rare, including in those patients with sporadic CD (27). This contrasts with findings in the sporadic counterparts to other tumors typical of the MEN1 syndrome, such as parathyroid adenomas (28–32) and gastrinomas (33, 34) in which somatic MEN1 gene mutation is very common.

## ACTH-independent Cushing’s syndrome in MEN1 due to adrenal disease

Besides functional tumors of pituitary corticotropes that may cause CS in MEN1, CS can also result from ACTH-independent causes originating in the adrenals. Adrenal gland enlargement and adrenal nodularity are very common in MEN1 (35–37). Although most adrenal lesions are non-functional, ACTH-independent CS due to benign or malignant adrenal tumors has been well-documented in MEN1 (14, 35, 38). Besides the more common benign adrenal tumors resulting in CS, adrenocortical carcinoma (ACC) has been previously reported in MEN1 patients with CS (4, 35, 38–40). For example, in a prospective series of 38 patients with proven germline MEN1 mutation, two had functional adrenal tumors resulting in CS and/or virilization: one had bilateral adenomas and one had ACC (35). In a retrospective analysis of MEN1 patients that included 19 patients with CS, three of 14 subjects with an identifiable cause for their CS (21%) had ACTH-independent CS due to adrenal tumors, two of these three from ACC (14). In general ACC is greater than 10-fold more common among MEN1 adrenal lesions than among sporadic adrenal incidentalomas (38).

ACTH-independent Cushing’s syndrome due to adrenal neoplasia can represent the presenting manifestation of MEN1. A previously healthy 16-year-old girl, lacking a known family history of MEN1, presented with progressive weight gain, hirsutism, acne, irregular menses, extensive skin striae, fatigue, and muscle weakness associated with a cortisol-producing adrenal adenoma, leading to identification of a frameshift mutation in MEN1 in the proband and multiple family members (41). A 43-year-old woman who presented with amenorrhea, hirsutism, and acne, associated with a large adrenal mass, was subsequently shown to have ACC, biochemical CS, and MEN1 (42).

Even though loss of heterozygosity at the MEN1 locus on chromosome 11q13 can be frequently documented in ACC, somatic mutation of the MEN1 gene in sporadic benign or malignant adrenocortical tumors appears to be rare (43–45). Widely metastatic ACC associated with somatic mutations in both TP53 and MEN1 and rapidly progressing CS has been reported however (46).

Less commonly, primary aldosteronism due to the presence of adrenocortical aldosteronoma has been documented in association with the MEN1 syndrome (38, 47, 48). In fact, cases of primary aldosteronism are more common among MEN1 adrenal lesions than among sporadic adrenal incidentalomas (38).

## Cushing’s syndrome due to ectopic ACTH secretion in MEN1

Another cause of ACTH-dependent CS in MEN1 patients, apart from ACTH-secreting pituitary tumors causing CD, are extrapituitary neuroendocrine tumors that secrete ACTH ectopically. Ectopic production of ACTH causing CS is a recognized paraneoplastic phenomenon that has been documented in association with a wide variety of endocrine and non-endocrine neoplasms and that presents significant diagnostic and therapeutic challenges (49). That ectopic production of ACTH causing CS is rare in MEN1 is supported by the absence of any such patient among the 90 cases of ectopic CS reported in a large 20-year retrospective review (50). Similarly, no MEN1 patients were among 12 cases with ectopic CS and thymic neuroendocrine tumors (NETs) who underwent surgical resection in another large retrospective study from a single institution (51).

In the context of MEN1, CS resulting from ectopic ACTH production has nevertheless been associated almost exclusively with the presence of thymic NETs (“thymic carcinoids”) (5–8). Thymic NETs have been found in some 1 to 8% of MEN1 patients in several large series and are a major cause of morbidity and mortality (52–56). Risk factors for the development of thymic NETs include male gender and a history of smoking (52, 54, 56).

MEN1-related thymic NETs, including those associated with the ectopic production of ACTH causing CS, can be aggressive in nature with a high potential for metastasis and are associated with an increased mortality (53, 57). Analysis of tumor tissue from a patient with MEN1 operated on twice for a thymic NET associated with the ectopic production of ACTH causing CS showed an initial Ki-67 labeling index of 5% but on re-operation the tumor was found to be invasive with a Ki-67 index of 30% (7). Another patient with germline mutation-proven MEN1 associated with thymic NET and ectopic CS was found at surgery to have an unresectable, invasive anterior mediastinal mass with a Ki-67 index of >50% (58). Although not well studied in the context of MEN1, such high Ki-67 indices and the increase in Ki-67 index noted on sequential operations likely represent the dominance over time of the more aggressive MEN1-null thymic neuroendocrine tumor clones. Thymectomy in a male MEN1 patient with a smoking history, a thymic NET, and ectopic CS revealed tumor invasion of the right atrium and metastases to the mediastinal lymph nodes- the patient eventually died from metastatic disease (5).

Rarely, CS from the ectopic production of ACTH from thymic NET can be the presenting manifestation of MEN1. Amoli and co-workers described a 29-year-old man who presented with psychosis due to ectopic CS associated with a large anterior mediastinal cystic mass with diffuse lymphadenopathy (59). After excision of a large, invasive, thymic NET, the patient experienced an initial period of remission, but CS recurred after 2 years, this time associated with primary hyperparathyroidism and hyperprolactinemia, and a clinical and genetic diagnosis of MEN1 was confirmed (59).

Thymic NET, including cases associated with CS due to the ectopic production of ACTH, can cluster in MEN1 families (54, 56, 60). Two of four siblings with MEN1 in Chinese kindred developed ectopic CS due to the production of ACTH from thymic NETs, and a third sibling with MEN1 had a thymic NET that stained positive for ACTH upon immunohistochemical analysis (8).

## Finding an etiology for Cushing’s syndrome in MEN1

Because of the potential clinical complexity in MEN1 and the fact that hypercortisolism can result from pituitary, adrenal, or other endocrine tumors, finding an etiology for MEN1-associated CS be challenging. In a retrospective series of 19 MEN1 patients with CS evaluated at one institution, an etiology could not be found in five patients, and among these the hypercortisolism appeared to resolve spontaneously in three (14). Al Brahim et al. describe an MEN1 patient with CS and two pituitary adenomas, one a corticotrope microadenoma, as well as ectopic production of corticotropin-releasing hormone from a thymic NET (17). Contributing to the diagnostic challenge is the recognition that periodic or cyclic CS may occur with all etiologies of CS (61).

## Discussion

CS is an uncommon manifestation of the autosomal dominant familial cancer predisposition syndrome MEN-1 that can be caused by both ACTH-dependent and independent mechanisms of hypercortisolism. It is therefore important to consider all etiologies for patients with MEN and CS, since hypercortisolism due to CD, adrenal etiologies, and the ectopic secretion of ACTH are well-documented in MEN1 just as in non-familial cases.

In MEN1, the presence of multiple synchronous or metachronous pituitary tumors may be discovered during pre-operative evaluation or surgery for CD, consistent with other “multiplicities” that characterize this familial tumor syndrome. Such potential pituitary tumor multifocality must be kept in mind during preoperative tumor localization for CD in MEN1 patients, since visualized pituitary lesions may not necessarily correspond to the culprit functional corticotrope adenoma.

Since both CD and ACTH-independent Cushing’s syndrome due to adrenal neoplasia can present in childhood or adolescence as its initial manifestation, MEN1 must be considered for all younger patients diagnosed with CS, especially in those with a potentially relevant family medical history. Early recognition of MEN1 in such patients could be beneficial by leading to earlier surveillance for other functional or non-functional endocrine and other tumors that might impact growth, development, and/or sexual maturation.

Even though most adrenal lesions are non-functional, adrenal gland enlargement and adrenal nodularity are very common in MEN1. Careful functional evaluation and determination of tumor imaging characteristics of adrenal lesions > 1 cm is important in MEN1, especially keeping in mind that ACC is > 10-fold more common among MEN1 adrenal lesions than among sporadic cases. That being the case, benign adrenal adenomas are still much more common among MEN1 patients with functional adrenal masses resulting in ACTH-independent CS.

Thymic NETs are the manifestation of MEN1 most often associated with the ectopic secretion of ACTH resulting in CS. Unfortunately, MEN1-related thymic NETs are frequently aggressive in nature with a high potential for metastasis and are associated with an increased mortality. This has led to recommendations for the performance of a prophylactic transcervical, near-total thymectomy concurrent with parathyroid surgery in MEN1 (62, 63), however strong data supporting the preventive efficacy of this intervention may be lacking (64). There have been rare reports of CS, extrapituitary in etiology but often difficult to distinguish from CD, resulting from ectopic production of corticotropin-releasing hormone (CRH), from pheochromocytomas, medullary thyroid cancers, and other neuroendocrine tumors (65–68). To date however, no such cases have been reported in the context of MEN1.

Because of the inherent endocrine complexity in many cases, it may not always be possible to determine the etiology of endogenous CS in MEN1. Yet we now have available advanced diagnostic tools that include bilateral inferior petrosal sinus sampling (69), optimized MRI protocols for pituitary lesions, such as fat-suppressed volumetric interpolated breath-hold examination (VIBE) imaging (70), high resolution computed tomography, functional imaging with somatostatin analogs and 18F-DOPA, and dynamic testing with desmopressin (71) and corticotrophin-releasing hormone. With this armamentarium, the etiology for most cases of CS in MEN1 should be determinable, especially when combined with careful observation and the clinical acumen of experienced medical endocrinologists, interventional radiologists, and endocrine surgeons. An even higher degree of diagnostic success may emerge from the future utilization of advanced artificial intelligence and machine learning algorithms, already being successfully applied in the field of endocrinology and metabolism (72, 73).

## Author contributions

WS conceived, organized, and wrote the manuscript.
